# Colocutaneous fistula as a consequence of titanium implant migration following sternal chondrosarcoma resection: a case report

**DOI:** 10.1093/jscr/rjag753

**Published:** 2026-08-30

**Authors:** Landrit Mehaj, Dafina Mahmutaj, Valon Zejnullahu, Vigan Shala, Oltion Braha

**Affiliations:** Department of Abdominal Surgery, University Clinical Center of Kosovo(QKUK), Rrethi i Spitalit p.n, Prishtina 10000, Kosovo; Department of Abdominal Surgery, University Clinical Center of Kosovo(QKUK), Rrethi i Spitalit p.n, Prishtina 10000, Kosovo; Department of Abdominal Surgery, University Clinical Center of Kosovo(QKUK), Rrethi i Spitalit p.n, Prishtina 10000, Kosovo; Department of Abdominal Surgery, University Clinical Center of Kosovo(QKUK), Rrethi i Spitalit p.n, Prishtina 10000, Kosovo; General Medicine, Faculty of Medicine, University of Prishtina, Bulevardi i Dëshmorëve p.n., Prishtina 10000, Kosovo

**Keywords:** sternal chondrosarcoma, titanium implant, implant migration, colocutaneous fistula, case report

## Abstract

Sternal chondrosarcoma is a rare malignancy requiring wide resection and chest wall reconstruction, typically using titanium implants. These implants may rarely lead to late migration. We report a 72-year-old male who presented with a colocutaneous fistula in 2026, 10 years after total sternectomy and titanium reconstruction for low-grade chondrosarcoma. He remained asymptomatic for 4 years, then developed skin erythema at the inferior edge of the surgical scar, which progressed to fecal discharge but delayed consultation for 4 years due to absence of systemic symptoms. Computed tomography showed migration of the titanium implant. Management included implant removal, extended right hemicolectomy, and ileo-transverse anastomosis. The chest wall was stabilized with a thoracic corset. This case demonstrates the need for prolonged clinical and radiological vigilance for late mechanical failure in long-term survivors of low-grade sternal tumors, emphasizing that subtle skin changes over the prosthesis should prompt early cross-sectional imaging to prevent severe visceral complications.

## Introduction

Primary sternal chondrosarcoma accounts for <1% of all chondrosarcomas [1]. The gold standard treatment is wide R0 resection, often requiring total sternectomy and complex chest wall reconstruction with titanium plates [2, 3]. Although titanium offers excellent biomechanical strength, late complications including hardware fracture, loosening, and migration occur in 2%–5% of cases, typically within the first 5 years [4, 5].

## Case report

**Figure 1:**
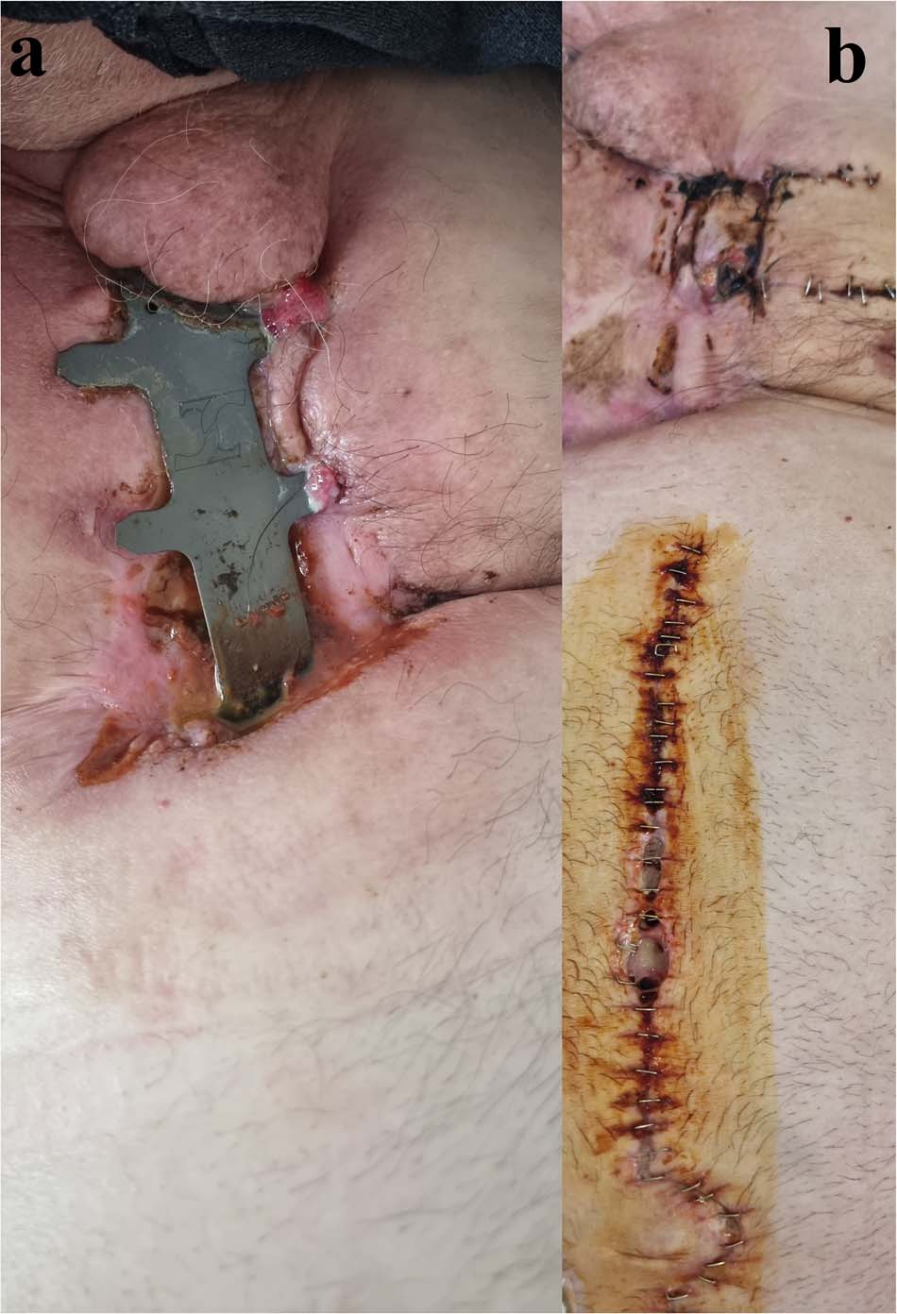
Preoperative and postoperative clinical images; (a) preoperative photograph showing the migrated metallic implant visible beneath the skin and fecaloid discharge from the fistulous opening in the epigastrium; (b) postoperative image showing the healed surgical wound after implant removal and right hemicolectomy.

In February 2026, a 72-year-old male presented to our department with a discharging fistula on the anterior abdominal wall (Fig. 1a). His medical history revealed that he had undergone total sternectomy abroad in December 2016 for low-grade chondrosarcoma, which was reconstructed with a titanium plate and fixator system. He had received no adjuvant radiotherapy.

**Figure 2:**
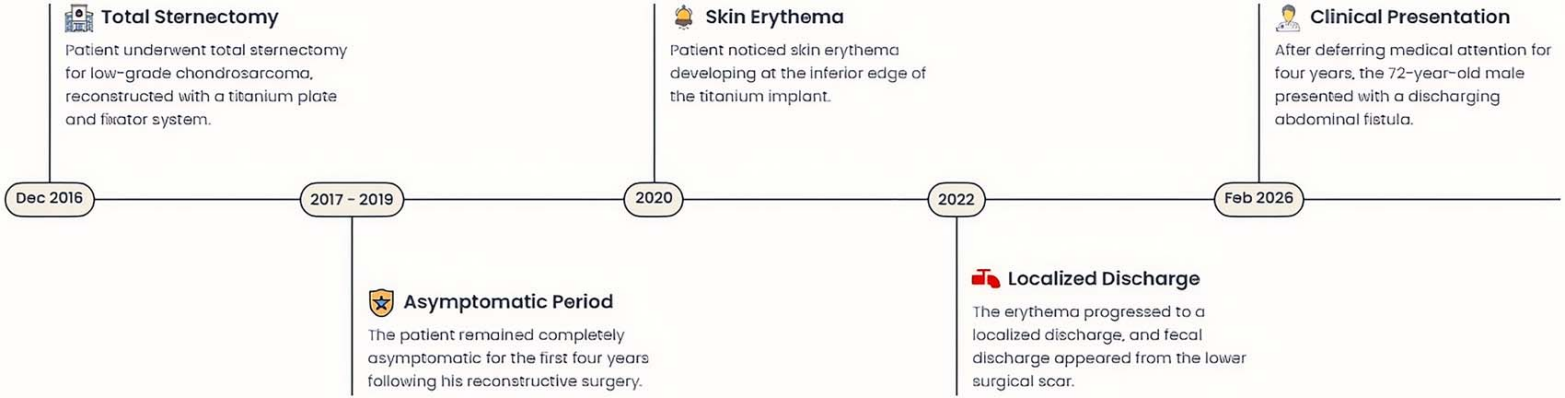
Timeline of clinical events from initial surgery to presentation.

He remained asymptomatic for the first 4 years following surgery. In 2020, he noticed skin erythema at the inferior edge of the implant. This progressed to a localized discharge by 2022. Although a fecal discharge from the lower surgical scar appeared in 2022, he deferred seeking medical attention for an additional 4 years, presenting to our department only in February 2026 (Fig. 2). He had remained systemically well throughout this period, which contributed to his delay.

**Figure 3:**
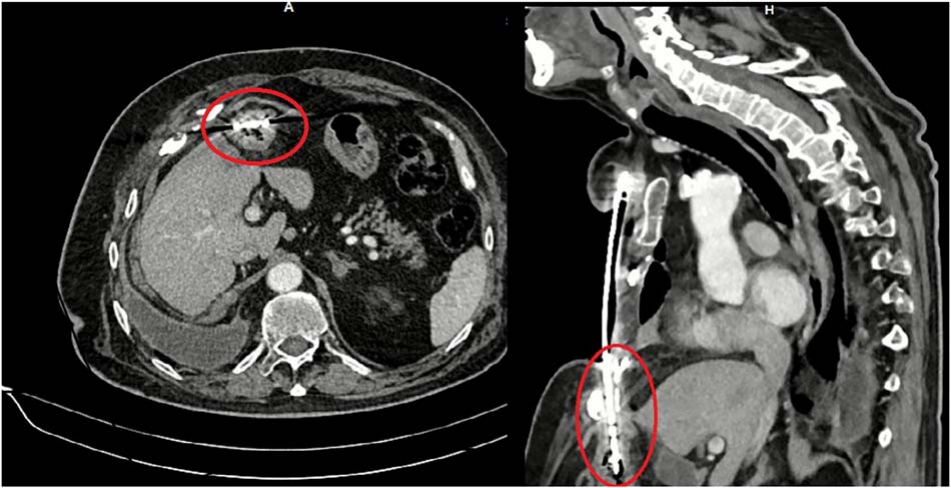
(Left) Axial CT view showing the high-density metallic hardware eroding into the transverse colon; (right) sagittal reconstruction illustrating the significant inferior migration of the titanium bar from the substernal space into the epigastric region.

Contrast-enhanced computed tomography (CT) demonstrated that the distal portion of the titanium implant had completely detached from the costal fixators and migrated inferiorly into the epigastric region, penetrating the transverse colon wall and forming a fistulous tract to the skin (Fig. 3).

**Figure 4:**
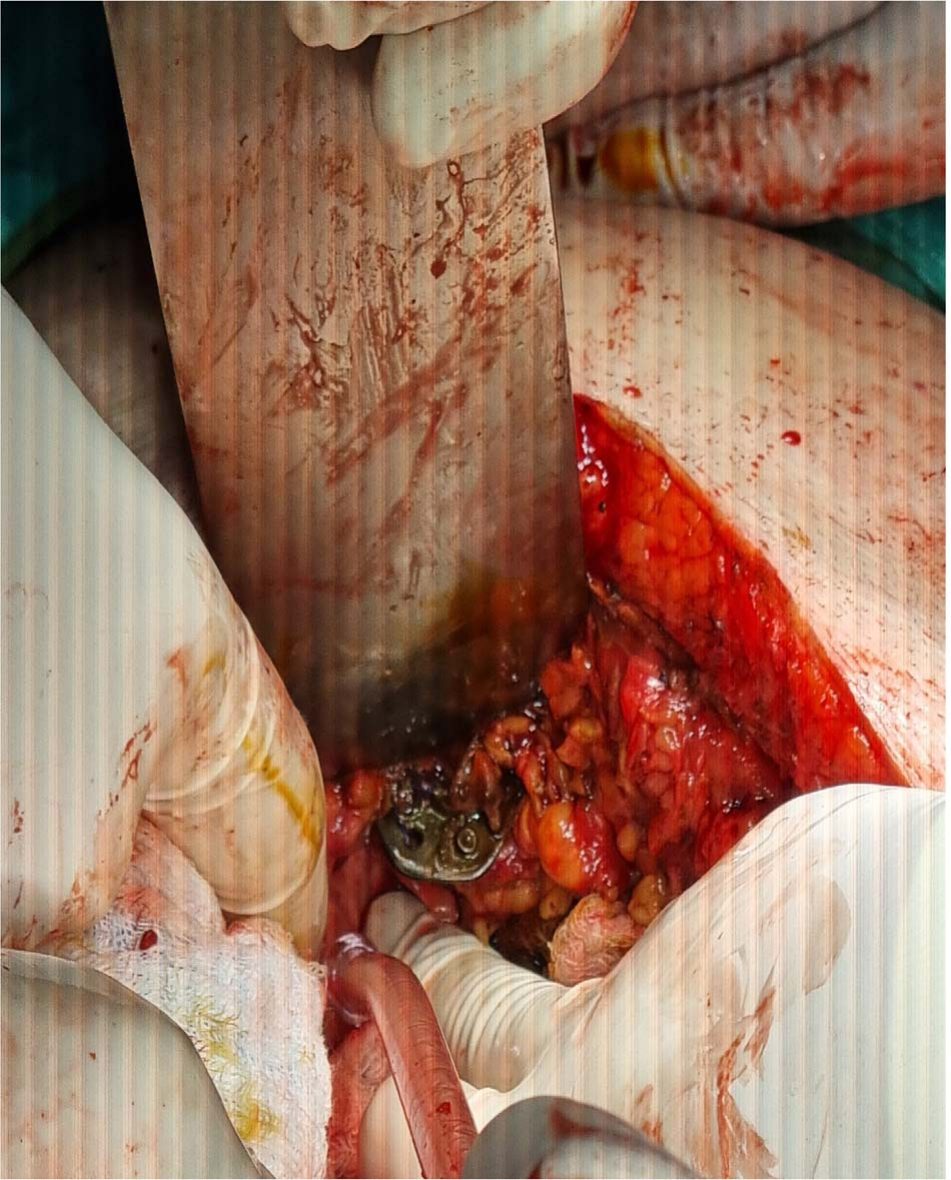
Intraoperative image showing the distal portion of the implant embedded in the greater omentum and eroding through the transverse colon wall.

The patient underwent exploratory laparotomy. Intraoperative findings confirmed that the migrated implant was embedded in the greater omentum and had caused full-thickness erosion of the transverse colon (Fig. 4). An extended right hemicolectomy was performed with implant removal and ileo-transverse anastomosis. The chest wall defect was stabilized using a custom-fitted thoracic corset.

The postoperative course was uneventful. He was discharged on the 10th postoperative day. At 3-month follow-up, the abdominal wall was healed, and he reported no respiratory compromise (Fig. 1b).

## Discussion

This case illustrates an exceptionally late complication of sternal reconstruction [6]. Continuous mechanical stress from respiratory motion can lead to hardware fatigue and fixator failure [7]. However, this case is remarkable for several reasons:

Firstly, no prior case of sternal hardware migration has been documented to result in a colocutaneous fistula. While hardware complications are well-recognized, visceral erosion with fistula formation represents an extreme and previously unreported consequence.

Secondly, our patient had never received adjuvant radiotherapy; thus, the transmural colonic injury was unequivocally caused by mechanical erosion alone, without the confounding effects of radiation-induced tissue fragility.

Thirdly, and most importantly, this complication occurred in a patient with a low-grade chondrosarcoma—a tumor with an excellent long-term prognosis, with 10-year survival rates reportedly exceeding 90% in selected series [1]. This “survival biology” argument is critical: patients with low-grade malignancies live long enough to manifest late prosthetic failures that would be clinically overshadowed by disease progression in higher-grade tumors. In this context, the absence of established guidelines for hardware surveillance represents a significant gap, as the risk of mechanical complications may persist for many years. A similar principle applies to other inert implants, where case reports document visceral erosion or fistula formation occurring decades after the index procedure. For instance, a hip implant was reported to have eroded into the terminal ileum, causing gastrointestinal bleeding 30 years after its placement [8]. Similarly, a cervical spine plate was reported to have migrated through the esophagus into the gastrointestinal tract 5 years after surgery [9]. These reports demonstrate that the risk of erosion and migration from hardware is a real and lasting concern, supporting the need for a high index of suspicion in this patient population.

The anatomical proximity of the proximal transverse colon to the lower thoracic aperture explains why the migrating sharp edge of the implant targeted this particular bowel segment [10]. The patient remained stable for 4 years with an active fistula due to a well-epithelialized tract that prevented free intra-abdominal spillage and peritonitis. This observation highlights that the absence of sepsis should not delay surgical exploration. A high index of suspicion is warranted whenever a patient with chest wall hardware presents with unexplained gastrointestinal symptoms or cutaneous changes over the prosthesis.

Complete removal of the migrated hardware combined with bowel resection and anastomosis was successful. We opted for a thoracic corset instead of immediate re-reconstruction with a new implant to avoid infection of prosthetic material in a contaminated field.

## Conclusion

Given the excellent long-term survival of patients with low-grade sternal chondrosarcoma, consideration should be given to extended clinical and radiological vigilance for late mechanical complications. While the optimal imaging interval remains undefined, we suggest that any new skin changes, erythema, or discharge over a chest wall prosthesis should prompt urgent cross-sectional imaging. This case highlights that the absence of systemic symptoms should not delay investigation in these patients, and a high index of suspicion for hardware-related complications must be maintained, particularly in this long-surviving patient population. Early recognition and timely surgical intervention are essential to prevent more serious complications.
